# Myotonic Myopathy With Secondary Joint and Skeletal Anomalies From the c.2386C>G, p.L796V Mutation in *SCN4A*

**DOI:** 10.3389/fneur.2020.00077

**Published:** 2020-02-13

**Authors:** Nathaniel Elia, Trystan Nault, Hugh J. McMillan, Gail E. Graham, Lijia Huang, Stephen C. Cannon

**Affiliations:** ^1^Department of Physiology, David Geffen School of Medicine at UCLA, Los Angeles, CA, United States; ^2^Molecular, Cellular, and Integrative Physiology Program, UCLA, Los Angeles, CA, United States; ^3^Division of Neurology, Children's Hospital of Eastern Ontario, University of Ottawa, Ottawa, ON, Canada; ^4^Department of Genetics, Children's Hospital of Eastern Ontario, University of Ottawa, Ottawa, ON, Canada

**Keywords:** skeletal muscle, channelopathy, sodium channel, Na_V_1.4, myotonia, voltage-clamp

## Abstract

The phenotypic spectrum associated with the skeletal muscle voltage-gated sodium channel gene (*SCN4A*) has expanded with advancements in genetic testing. Autosomal dominant *SCN4A* mutations were first linked to hyperkalemic periodic paralysis, then subsequently included paramyotonia congenita, several variants of myotonia, and finally hypokalemic periodic paralysis. Biallelic recessive mutations were later identified in myasthenic myopathy and in infants showing a severe congenital myopathy with hypotonia. We report a patient with a pathogenic *de novo SCN4A* variant, c.2386C>G p.L796V at a highly conserved leucine. The phenotype was manifest at birth with arthrogryposis multiplex congenita, severe episodes of bronchospasm that responded immediately to carbamazepine therapy, and electromyographic evidence of widespread myotonia. Another *de novo* case of p.L796V has been reported with hip dysplasia, scoliosis, myopathy, and later paramyotonia. Expression studies of L796V mutant channels showed predominantly gain-of-function changes, that included defects of slow inactivation. Computer simulations of muscle excitability reveal a strong predisposition to myotonia with exceptionally prolonged bursts of discharges, when the L796V defects are included. We propose L796V is a pathogenic variant, that along with other cases in the literature, defines a new dominant *SCN4A* disorder of myotonic myopathy with secondary congenital joint and skeletal involvement.

## Introduction

Sodium channels are required for action potential generation and propagation. The sodium channel alpha-subunit gene (*SCN4A*) encodes the isoform Na_V_1.4 which is the most abundant isoform in skeletal muscle.

Mutations affecting one *SCN4A* allele are often associated with a gain-of-function resulting in muscle stiffness (myotonia) that may worsen with repeated contraction (paramyotonia) as well as episodes of hyperkalemic periodic paralysis (1). Phenotypic variability has been reported among family members regarding the age of onset and clinical severity of myotonia (2). Another class of heterozygous *SCN4A* mutations allow an anomalous leakage of ions through the voltage sensor of the channel, distinct from the sodium-conducting pore, and cause hypokalemic periodic paralysis (3).

With increased accessibility to genetic testing, the phenotypic spectrum of *SCN4A* mutations has been expanding. We now recognize *SCN4A* mutations to cause isolated exercise- or cold-induced myalgia (4). *De novo* heterozygous *SCN4A* mutations, often at p.Gly1306Glu, have been linked to a severe neonatal phenotype with episodic laryngospasm (5–8), stridor (9), or in other cases with apneic episodes (10).

More recently, biallelic mutations in *SCN4A* have been reported in congenital myasthenic syndromes (11–13), at times with myopathy (14), and in severe congenital myopathy with fetal hypokinesia (15). All these syndromes have recessive inheritance, with a single loss-of-function allele being asymptomatic, including even a functional null from a premature stop. Moderate loss-of-function mutations from enhanced inactivation are associated with congenital myasthenia (11), whereas biallelic mutations that include a single null cause congenital myopathy with fetal hypokinesia and biallelic null mutations are embryonic or neonatal lethal (15).

We report a patient who presented with arthrogryposis multiplex congenita, congenital myopathy, and episodes of bronchospasm who has the c.2386C>G, p.L796V variant in *SCN4A*. Expression studies of L796V channels revealed a two forms of gain-of-function, enhanced activation and impaired slow inactivation, and which in model simulations led to prolonged bursts of myotonic discharges. We propose L796V is a pathogenic mutation and that the clinical features shared with previously described cases defines a new *SCN4A* syndrome of myotonic myopathy with secondary deformities of joints and bone.

## Methods And Materials

### Sodium Channel Currents

Sodium currents were measured from fibroblasts (HEK cells) transiently transfected with plasmids encoding the wild type (WT) or mutant L796V human Na_V_1.4 α subunit and the β1 accessory subunit as previously described (16). Currents were recorded in whole-cell mode with a patch electrode that contained in mM: 100 CsF, 35 NaCl, 5 EGTA, 10 HEPES, pH to 7.3 with CsOH. The extracellular bath contained in mM: 140 NaCl, 4 KCl, 2 CaCl_2_, 1 MgCl_2_, 2.5 glucose, 10 HEPES, pH to 7.3 with NaOH. Cells with maximal peak Na^+^ currents <1 nA were excluded to minimize the contribution from endogenous Na^+^ currents (typically < 0.1 nA), and cells with peak Na^+^ currents >5 nA were excluded to avoid series resistance errors.

**Figure 1:**
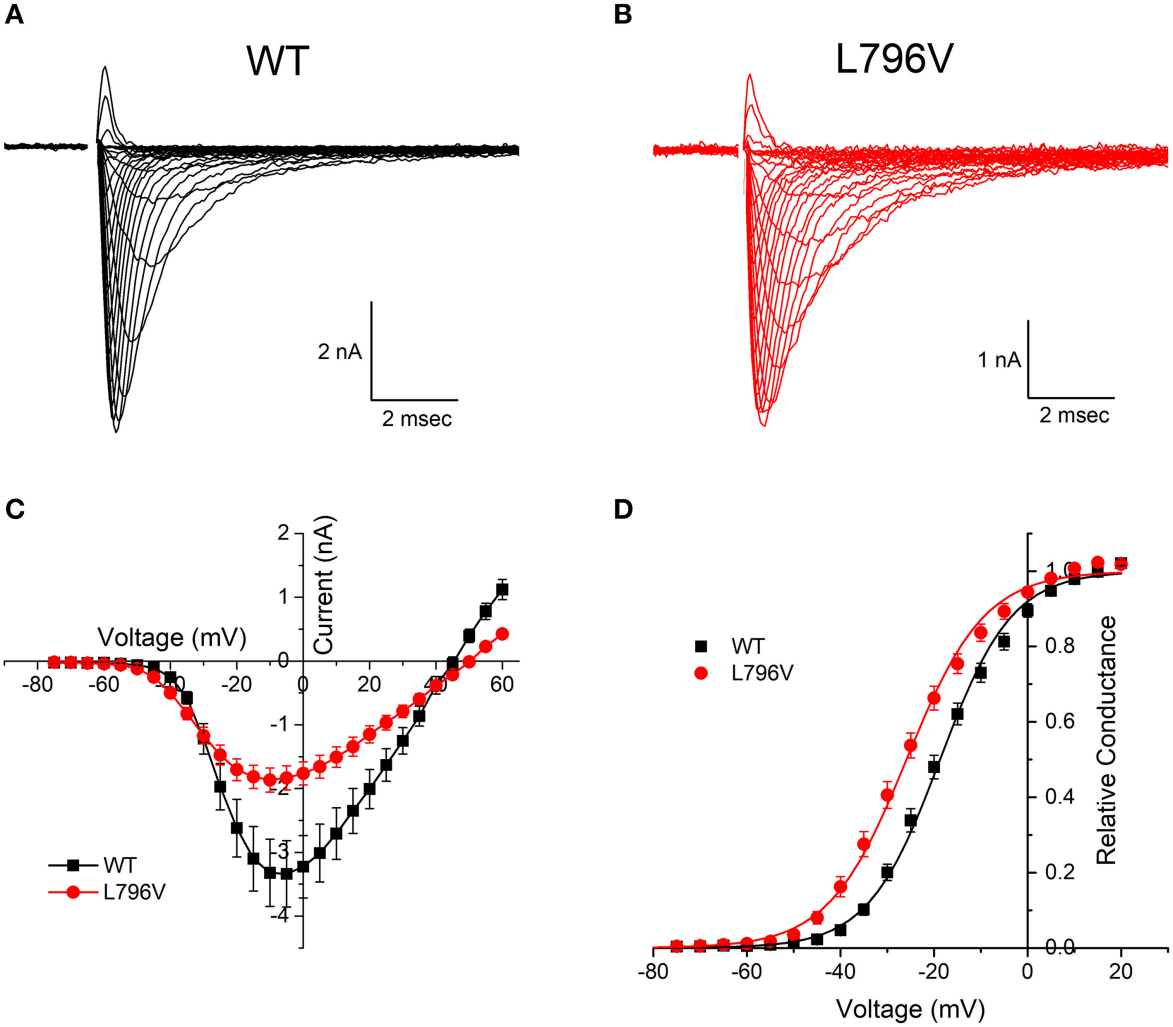
Activation of L796V channels is shifted toward more negative potentials. Sodium currents were recorded from HEK cells expressing WT **(A)** and L796V **(B)** channels. Superimposed traces show currents elicited by depolarization to test potentials of −75 to +60 mV from a holding potential of −120 mV. **(C)** Peak sodium current is shown as a function of test potential and reveals a reduced amplitude for L796V compared to WT. **(D)** Transforming the peak current to relative conductance (see Methods and Materials) shows a 7.2 mV hyperpolarized shift for L796V channels. Symbols show means from *n* = 16 (WT) or *n* = 10 (L796V) cells.

The voltage-dependent activation of sodium currents was quantified by fitting the peak amplitude (*I*_*peak*_) to a linear conductance (*G*_max_) with a reversal potential (*E*_*rev*_) that was scaled with a Boltzmann function: $I_{peak}=G_{\max}\left(V-\;E_{rev}\right)/\left(1+e^{-\left(V-\;V_{1/2}\right)/K}\right)$. The voltage-dependence for activation of the channel is reflected by *V*_1/2_, the voltage at which half the channels are activated, and *K* a steepness factor. The voltage dependence of the relative conductance (see Figure 1D) was calculated as *I*_*peak*_ divided by *G*_max_(*V* − *E*_*rev*_). The time constant, τ, for entry to inactivation was estimated from a single exponential fit of the current decay (fast inactivation) or of the change in peak current after progressively longer conditioning pulses (slow inactivation). The voltage dependence of steady-state fast inactivation was quantified by fitting the relative peak current after a 300 ms conditioning pulse at a voltage of *V*_*cond*_ by a Boltzmann function *I*_*peak*_(*V*_*cond*_)/*I*_*peak* max_ = 1/(1 + *e*^(*V−V*_1/2_)/K^). For the steady-state voltage dependence of slow inactivation, a plateau term (*S*_0_) was included because slow inactivation does not reduce channel availability to 0 at strongly depolarized potentials (Figure 3D). Estimated values for parameters are presented as mean ± SEM.

### Simulated Muscle Action Potentials

The functional consequences of altered sodium currents observed for the L796V mutant channels on muscle excitability were explored using computer simulation. The two-compartment model of a muscle fiber [modified from (17)] consisted of the plasma membrane (sarcolemma) and the transverse tubular membrane, each of which contained voltage-dependent conductances to simulate sodium channels, chloride channels, inward-rectifying potassium channels, and delayed-rectifier potassium channels. Mutant L796V channels were simulated by modifying the parameter values so that the currents predicted by a Hodgkin-Huxley model matched the currents we measured in the HEK cell expression system (see Supplementary Material for details).

## Results

### Clinical Presentation and Genetic Analysis

A 7 year old boy of Morrocan descent was the first child to healthy, non-consanguineous parents. Routine antenatal ultrasounds at 13, 21, and 30 weeks gestation revealed normal fetal anatomy and amniotic fluid levels. Good fetal movements were reported throughout gestation. Repeat ultrasound at 41 weeks showed decreased amniotic fluid levels prompting a planned Caesarian section. Apgar scores were 1 at 1 min, 5 at 5 min, and 7 at 10 min. Although no chest compressions were required, he was intubated at birth and empiric surfactant was administered. His birth weight was 2,995 g (3–10%ile) and head circumference 36.0 cm (50%ile). He was noted to have facial weakness and arthrogryposis multiplex congenita with extensive bilateral contractures at the elbows, wrists, fingers, hips, knees and ankles. He did demonstrate antigravity movements of his arms and legs. Nerve conduction studies at 2 weeks of age revealed normal median and medial plantar nerve sensory responses. Median and tibial motor responses revealed abnormally low compound motor action potential (CMAP) amplitude. Needle EMG of his biceps, vastus lateralis, gastrocnemius and abductor hallicus revealed increased insertional activity (Supplementary Video 1).

Beginning at 2 weeks old, he began having episodes of bronchospasm associated with cessation of chest movement and desaturation. Episodes lasted for 30 s to 2 min and showed minimal response to supplemental oxygen and positive pressure ventilation. Video EEG was unremarkable with no epileptiform activity noted at the time of clinical events.

MRI brain, echocardiogram, serum creatine kinase, and quadriceps muscle biopsy were unremarkable. Repeat EMG of the deltoid, first dorsal interosseous and tibialis anterior at 6 weeks of age again revealed increased insertional activity; however, there were long runs of myotonic discharges with typical waxing and waning variation in frequency and amplitude (Supplementary Video 2) that were not seen on the earlier study.

Although the bronchospasm did not show any appreciable response to phenobarbital, it abruptly stopped with empiric carbamazepine 20 mg/kg/day. He remains on carbamazepine. At 5 years of age he has reported some cold-induced episodes of eyelid and facial muscle paramyotonia but no episodes of periodic paralysis.

The patient's medical history was also relevant for severe gastroesophageal reflux due to a sliding hiatus hernia. He required a fundoplication and gastro-jejunostomy (GJ) tube placement (removed at 4 years old). He had strabismus which required surgical correction at 16 months of age. He had bilateral cryptorchidism requiring orchidopexy. He required multiple orthopedic surgeries including bilateral ankle casting and eventual contracture release (at 15 months); treatment of the developmental dysplasia of the left hip with open reduction (at 3 years).

His language development has progressed normally. He is fluent in three languages and requires no modification to his academic curriculum. Gross motor development was significantly delayed; he rolled independently at 6 months and sat independently at 18 months. At 2–1/2 years he was able to walk using a walker. At 3–1/2 years he was able to rise and walk independently.

Array CGH and molecular testing for myotonic dystrophy type 1 were normal. HSPG2 gene sequencing revealed that the patient was heterozygous for a G>A transition in a highly conserved residue (nucleotide 4877, exon 39) (seen in 0.8% of the population) that had not been reported as either a mutation or a polymorphism. MLPA testing revealed no deletion or duplication affecting the other allele and immunohistochemical staining for perlecan on frozen muscle from prior biopsy was normal. LIFR sequencing and deletion-duplication analysis was normal. Collectively, these genetic and histochemical findings do not support a diagnosis of Schwartz-Jampel syndrome. Exome sequencing, performed as part of the Canada-wide Care4Rare research consortium identified a likely pathogenic variant in one *SCN4A* allele that was confirmed with Sanger sequencing: SCN4A: NM_000334: c.2386C>G, p.Leu796Val. It was not present in either parent.

### Functional Characterization of L796V Mutant Sodium Channels

#### Activation of L796V Channels Was Shifted to More Negative Potentials

Both wild type (WT) and L796V mutant sodium channels were expressed in the plasma membrane of transiently transfected fibroblasts (HEK cells), as shown in Figure 1 by the sodium currents recorded in whole-cell voltage clamp. A plot of the peak sodium current as a function of membrane voltage (Figure 1C) shows the current amplitude was lower for cells expressing L796V compared to WT (−2.07 ± 0.19 and −3.32 ± 0.53 nA, respectively). A more accurate comparison of expression level is obtained from the maximum conductance, *G*_max_, which is reflected by the slope of the current-voltage relation at voltages > 20 mV. On average, the *G*_max_ was 50% lower for cells expressing L796V than WT channels (37.4 ± 3.5 and 76.4 ± 10 nS, respectively, *p* < 0.05).

The voltage-dependence of activation is illustrated more clearly by transforming the peak current amplitude to relative conductance as a function of test potential (see section Methods and Materials), as shown in Figure 1D. The midpoint of the relative conductance curve, *V*_1/2_, was shifted toward more negative potentials by −7.2 mV L796V channels (−25.9 ± 1.3 mV, *n* = 10) compared to WT channels (−18.7 ± 1.1 mV, *n* = 16; *p* < 0.001). The voltage dependence was slightly less steep for L796V channels (8.32 ± 0.20 mV) compared to WT (7.31 ± 0.22), but these values were not statistically different at the 0.05 level.

#### Fast Inactivation Was Not Altered by the L706V Mutation

**Figure 2:**
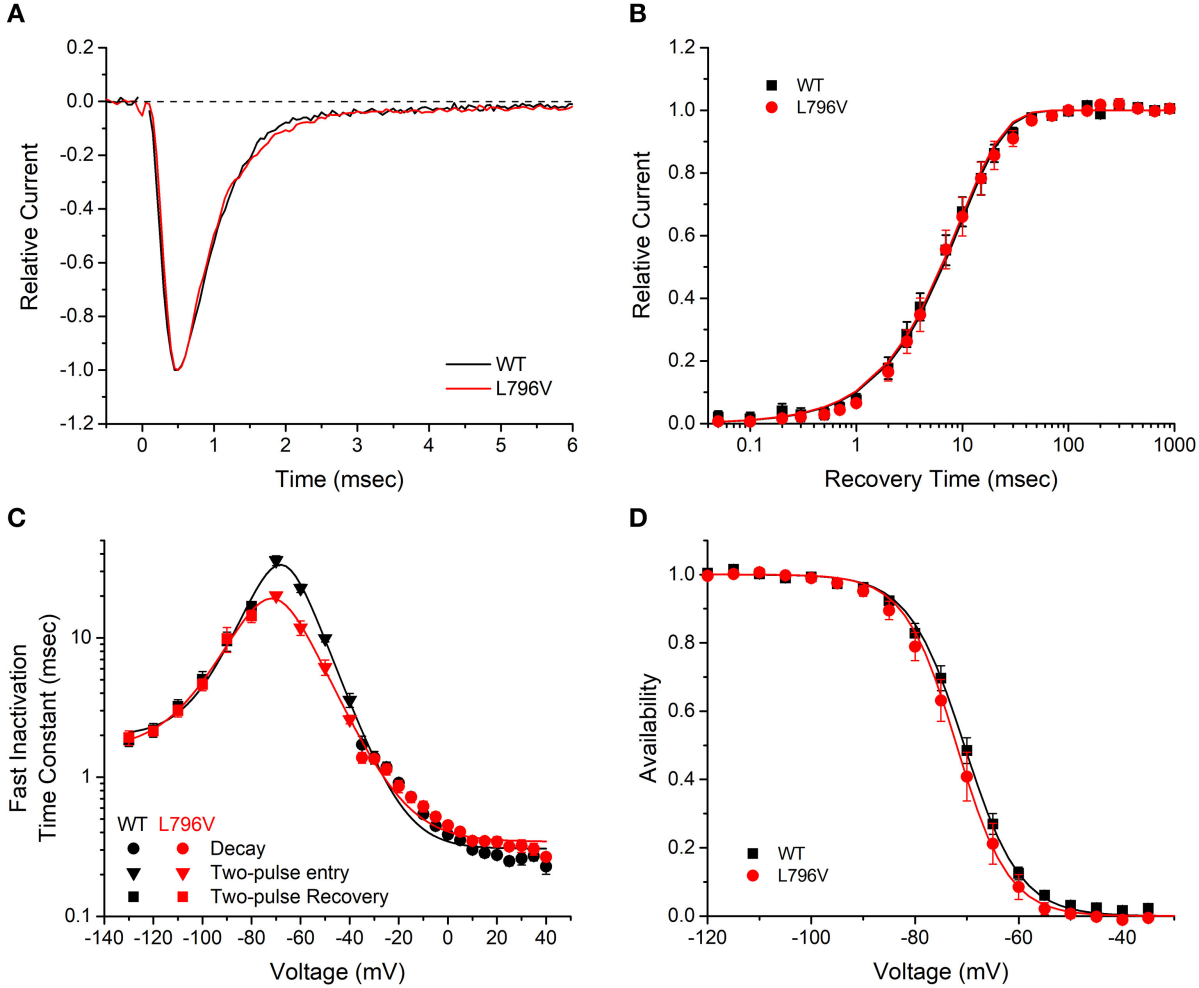
Fast inactivation is not affected by the L796V mutation. **(A)** Sodium current decay kinetics are indistinguishable for WT and L796V channels. Representative current traces elicited at −10 mV have been normalized by peak amplitude. **(B)** Recovery from fast inactivation is identical for WT and L796V channels. Data show the time course for the recovery of peak current amplitude at a holding potential of −90 mV, after channels were inactivated with a conditioning pulse of 30 ms at −10 mV. Symbols show means for WT (*n* = 8) and L796V (*n* = 5). **(C)** Summary plot of the time constants for entry or recovery from fast inactivation shows identical kinetics for WT and L796V channels. Three separate protocols were used to measure inactivation kinetics, depending on the voltage range, as described in section Methods and Materials. Symbols show means from WT (*n* = 16) and L796V (*n* = 10). **(D)** The steady-state voltage dependence of fast inactivation was indistinguishable for WT (*n* = 15) and L796V (*n* = 10) channels. Plot shows relative amplitude for the peak sodium current elicited by a depolarization to −10 mV after a 300 ms conditioning pulse at the indicated voltage (abscissa).

Several voltage-pulse protocols were used to characterize the kinetics and steady-state voltage dependence of sodium channel fast inactivation. The time constant of the sodium current decay after the early peak provides a quantitative measure of channel fast inactivation from the open state. Superposition of amplitude-normalized current traces (Figure 2A) shows the fast inactivation time course was indistinguishable between WT and L796V channels, and the values of the time constants over the measurable range from −35 to + 40 mV were overlapping (Figure 2C, circles). Recovery from fast inactivation was measured in a two-pulse protocol. First, channels were fast inactivated by a 30 ms conditioning pulse to −10 mV, then after a variable recovery interval at a hyperpolarized potential, the amount of recovery was measured as the relative current elicited by a test pulse to −10 mV. The time course of recovery at −90 mV was indistinguishable for WT and L796V channels (Figure 2B), and the time constant for recovery was identical over the measured range from −130 to −80 mV (Figure 2C, squares). The kinetics of entry to fast inactivation from closed states was measured in a two-pulse protocol for which a variable duration conditioning pulse was applied to partially inactivate channels (without opening), and then a test pulse to −10 mV was applied to measure the relative current. The time constant for the onset of close-state fast inactivation was modestly smaller for L796V over the voltage range from −70 to −50 mV (Figure 2C, inverted triangles). This difference is not predicted to be biologically significant, because the more rapid kinetics of entry to inactivation at the peak of the action potential (0–20 mV) and the rapid recovery at the resting potential (−80 to −95 mV) will dominate the kinetics of fast inactivation in muscle fibers. The voltage dependence of steady-state fast inactivation was measured as the relative peak current elicited at −10 mV, after a 300 ms conditioning pulse to potentials over a range from −120 to −40 mV. The data were indistinguishable for WT and L796V channels (Figure 2D), as confirmed by the parameter estimates from fits to a Boltzmann function (*V*_1/2_ −70.5 ± 0.91 mV *n* = 15, −72.0 ± 1.5 mV *n* = 10 for WT and L796V, respectively; *K* 5.37 ± 0.56 mV, 5.02 ± 0.40 mV for WT and L796V, respectively).

#### Slow Inactivation Was Impaired by L796V

**Figure 3:**
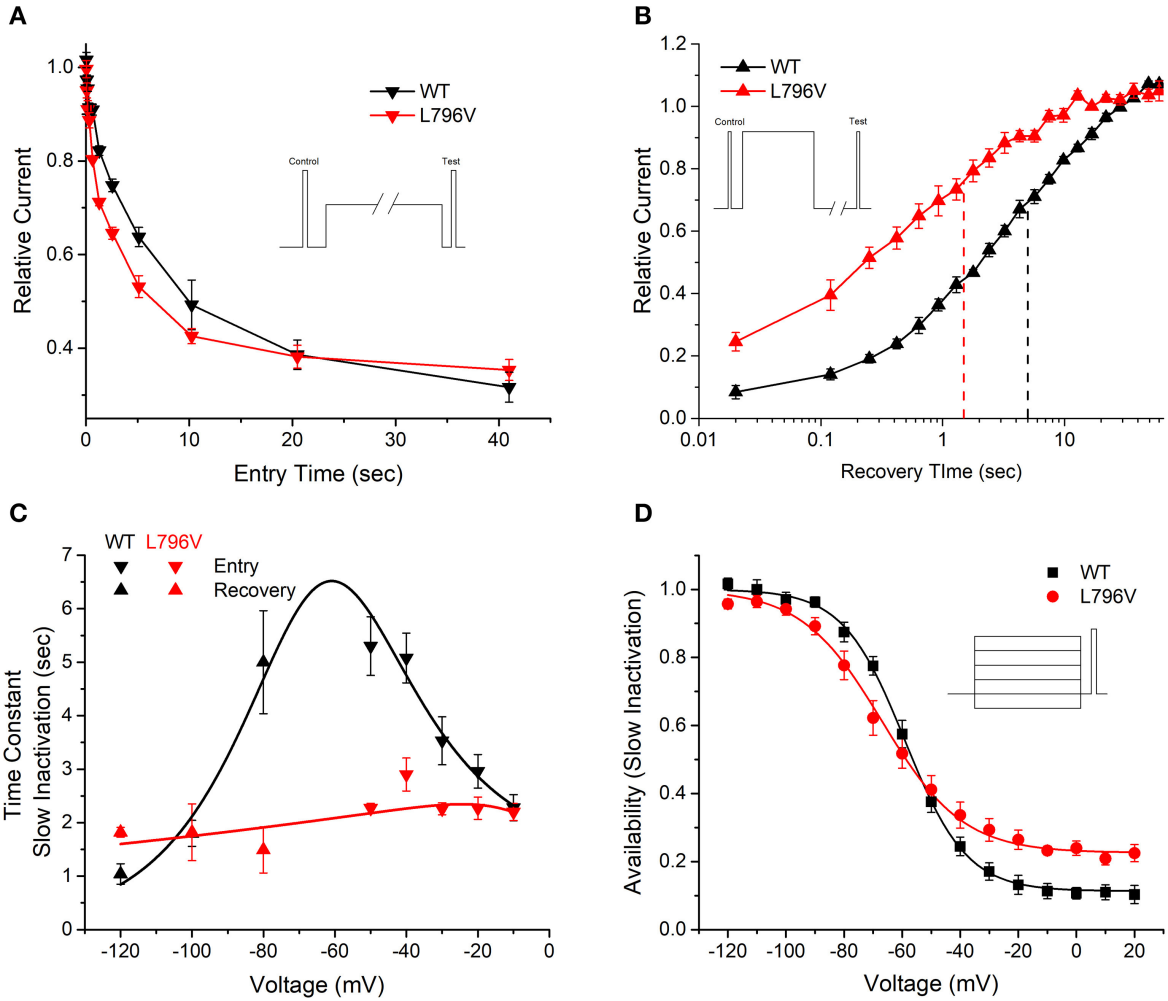
Slow inactivation is altered by the L796V mutation. **(A)** A two-pulse protocol with a variable duration condition pulse to −50 mV (inset) shows accelerated onset of slow inactivation for L796V channels. Symbols show means for WT (*n* = 5) and L796V (*n* = 3) channels. **(B)** Recovery from slow inactivation at −80 mV is accelerated four-fold for L796V compared to WT channels. Channels were slow inactivated with a 30 s conditioning pulse to −10 mV (inset), and then recovery from slow inactivation was measured as the relative peak current elicited by a brief pulse to −10 mV, after variable period of recovery at −80 mV. Symbols show means for WT (*n* = 5) and L796V (*n* = 4). **(C)** Summary plot for the kinetics of slow inactivation entry and recovery shows a nearly voltage-independent time constant for L796V mutant channels. Symbols show means for WT (*n* = 5) and L796V (*n* = 4) channels. **(D)** Steady-state voltage dependence of slow inactivation shows a decreased slope (−100 to −40 mV range) and less complete slow inactivation (higher plateau at **–**20 to +20 mV) for L796V (*n* = 5) compared to WT (*n* = 5) channels.

Slow inactivation of sodium channels occurs on a time scale of seconds, compared to the millisecond range for fast inactivation. Depolarization promotes both forms of inactivation, and the slow inactivated component is experimentally resolved by measuring the proportion of current that fails to recover during a brief hyperpolarization (−120 mV for 20 ms). The time course for the onset of slow inactivation is shown in Figure 3A, in which repeated trials with a progressively longer duration conditioning pulse to −50 mV have been applied and the relative current decreased as a greater proportion of channels became slow inactivated. The time constant of this exponential decay is shown for various test potentials from −50 to −10 mV in Figure 3C (inverted triangles). The time constant decreases (faster entry rate) at more positive potentials for WT channels, whereas the time constant is voltage independent and small for L796V channels.

The time course for recovery from slow inactivation at hyperpolarized potentials was measured by monitoring the recovery of peak sodium current after a 30 s conditioning pulse to −10 mV to maximally slow inactivate channels. Recovery at −80 mV is shown in Figure 3B. Fewer L796V channels are slow inactivated at the beginning of the recovery period (25% current already recovered at 0.020 s compared to 10% for WT) and the time course of recovery of L796V is more rapid than for WT (left shift of recovery curve). The recovery time constant measured over a voltage range from −80 to −120 mV was independent of voltage for L796V channels (Figure 3C, upright red triangles), whereas for WT channels the time constant decreased (faster recovery) with hyperpolarization (Figure 3C, upright black triangles).

The voltage dependence of steady-state slow inactivation was measured as the loss of current availability (i.e., decreased peak current amplitude) measured after a 30 s conditioning pulse (Figure 3D). The maximal extent of slow inactivation was reduced for L796V channels, as shown by the higher amplitude plateau in the availability curve at voltages more positive than −20 mV. The fitted parameter estimates for the plateau were *S*_0_ 0.11 ± 0.07, *n* = 5; 0.23 ± 0.02, *n* = 5 for WT and L796V, respectively (*p* = 0.01). In addition, the steepness of the voltage dependence was reduced for L796V channels (*K* 10.7 ± 1.0 mV, *n* = 5 for WT; 13.9 ± 0.4 mV, *n* = 5 for L796V, *p* < 0.01). There was a trend for a hyperpolarized shift in the midpoint for the voltage dependence of slow inactivation for L796V, but this difference was not distinguishable statistically (*V*_1/2_ −59.1 ± 1.9 mV, *n* = 5 for WT; −67.0 ± 3.4 mV, *n* = 5 for L796V, *p* = 0.11).

**Figure 4:**
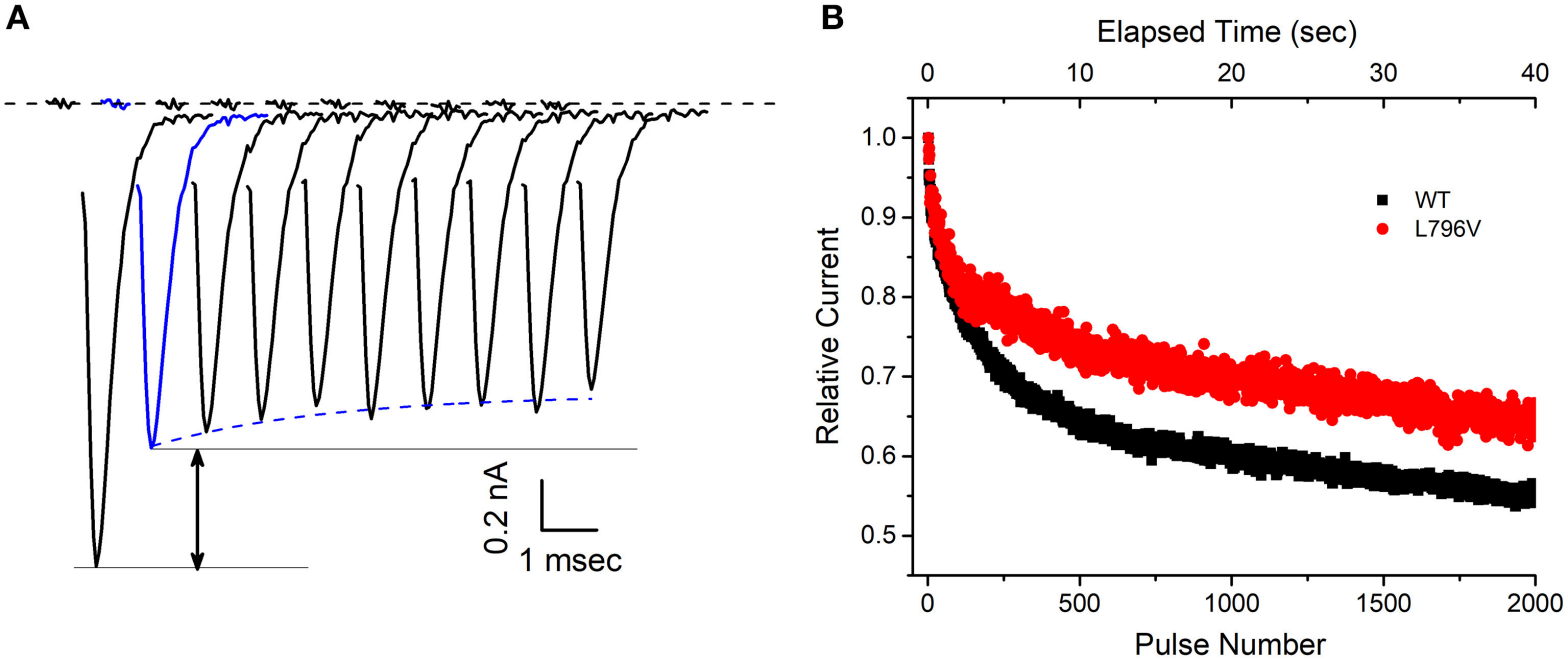
The decline of sodium current peak amplitudes during a 50 Hz train of pulses is less pronounced for L796V channels, thereby showing a gain-of-function change caused by altered slow inactivation. **(A)** Exemplary WT sodium currents elicited by the first 10 pulses in a 50 Hz train of 3 ms depolarizations to −10 mV are shifted in time and superimposed to illustrate the decline in peak amplitude. The holding potential between pulses was −80 mV. The large decline between the first and second pulse (black arrow) reflects trapping of channels in the fast-inactivated state. The subsequent decline starting from the second pulse (blue) is caused by loss of channels successively trapped in the slow-inactivated state. **(B)** The decline in peak sodium current during a prolonged 50 Hz train of 3 ms pulses is attenuated for L796V channels. Points show mean relative current for WT (*n* = 7) and L796V (*n* = 3) channels, as defined by normalization to the peak amplitude of the sodium current elicited by the second pulse (blue).

At first glance, the changes in slow inactivation properties for L796V channels appear to be a mixture of gain and loss of function effects. Enhancement of slow inactivation is expected at the resting potential of −80 mV because of the reduced slope of the voltage dependence and the tendency for a left shift (Figure 3D, reduced availability at −80 mV), as well as for a faster rate of entry over the voltage range of −50 to −30 mV (Figure 3C, smaller time constants). On the other hand, impairment of slow inactivation is expected at depolarized potentials because inactivation of L796V is less complete than WT (Figure 3D, higher plateau −20 to 20 mV), and the recovery from slow inactivation is faster for L796V at the resting potential (Figure 3C, smaller time constant at −80 mV). We propose the overall effect will be impaired slow inactivation for L796V channels, in the context of the slow inactivation that occurs during sustained bursts of action potentials (e.g., myotonia). The basis for this prediction is that entry to slow inactivation occurs primarily at voltages near the peak depolarization of the action potential (where the predominant change is less complete slow inaction for L796V) and trapping of channels in the slow inactivated state is primarily dependent on the rate of recovery at the resting potential of −80 mV (which is faster for L796V channels). This prediction is supported by experimental evidence showing the use-dependent reduction of sodium current is more pronounced for WT than L796V channels during repetitive stimulation at 50 Hz. Figure 4A shows a superposition of sodium currents recorded in response to the first 10 pulses to +10 mV from a holding potential of −80 mV. The initial decline in peak amplitude from the first to the second pulse is predominantly caused by incomplete recovery from fast inactivation, whereas the subsequent decline for additional pulses is due to progressive loss of channel availability from slow inactivation. The slow inactivation effect is illustrated in Figure 4B for the entire 40 second train of 3 ms depolarizations at 50 Hz (2,000 pulses). The peak amplitude for each pulse is normalized by the amplitude of the second pulse (Figure 4A, blue trace) to isolate the effect of slow inactivation, which under these conditions is about 10% less for L796V channels compared to WT.

#### Functional Defects of L796V Mutant Channels Cause Myotonia in a Simulated Muscle Fiber

**Figure 5:**
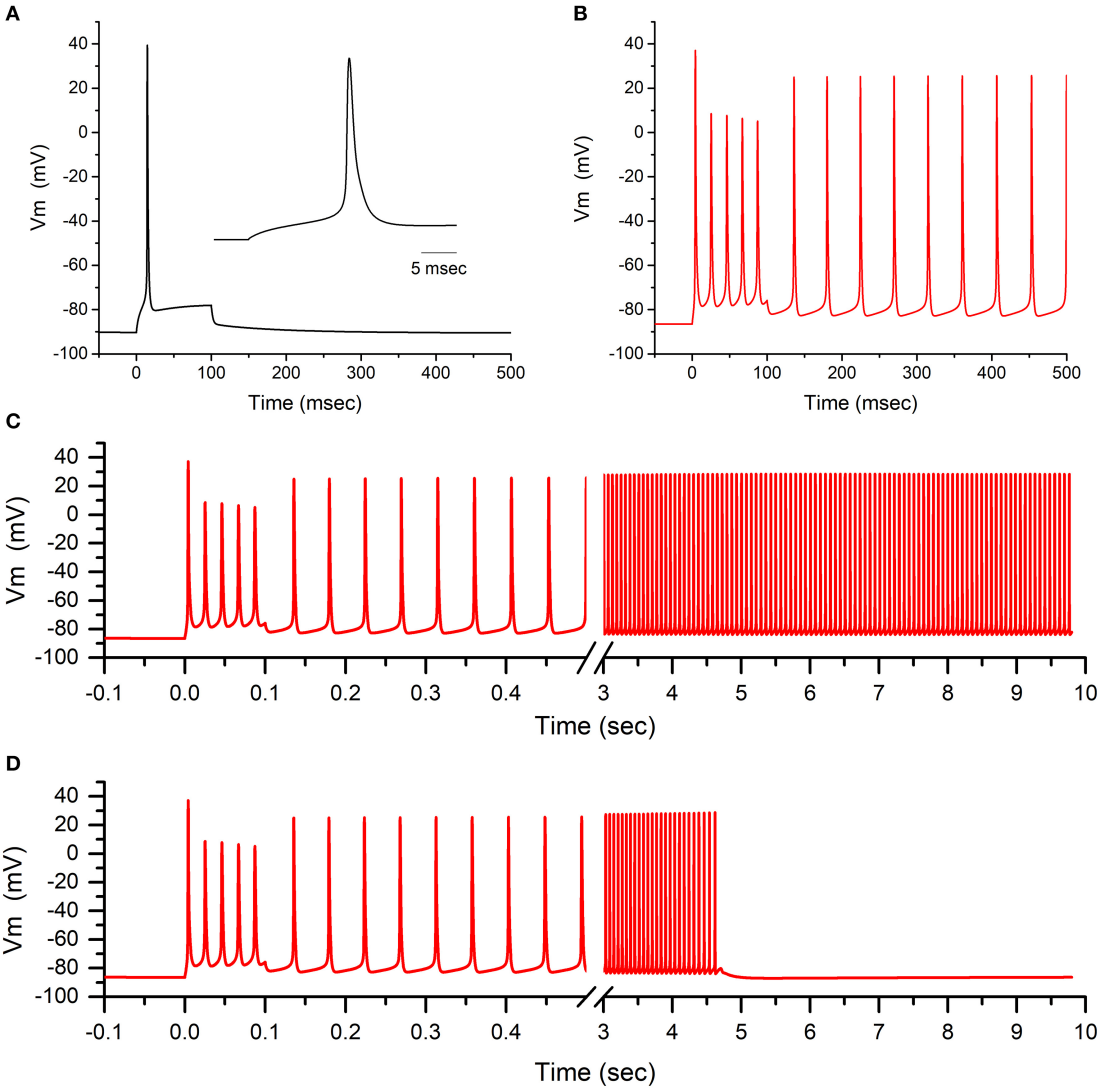
Model simulation predicts sustained busts of myotonia from the L796V channel defects. **(A)** The action potential elicited by a 20 μA/cm^2^ current pulse applied for 100 ms is shown for a simulated muscle fiber with normal values for voltage-activated ion channels. Inset shows the initial 25 ms of the model simulation. **(B)** When 50% of the simulated sodium channels are modeled using parameters to emulate the altered behavior of L796V channels, then the same 100 ms current stimulus triggers a burst of myotonic discharges that persist beyond the 100 ms duration of the stimulus. **(C)** Extended simulation over time shows stable self-sustained repetitive myotonic discharges that do not cease. **(D)** When the simulated mutant channels are modified to have the slow inactivation kinetics for WT channels, then use-dependent reduction of the sodium current is enhanced (see this figure) and the myotonic burst ends after 4.5 s.

The functional consequences of the defects in activation and in slow inactivation for L796V mutant channels were explored in a computational model of a muscle fiber (see Supplementary Material for details). For a simulated fiber with WT sodium channels, the resting potential was −90.3 mV and the voltage threshold to elicit an action potential was −66 mV. Susceptibility to myotonic discharges was tested by simulated injection of a 100 ms depolarizing current pulse (20 μA/cm^2^). A single action potential was elicited in a simulated WT fiber (Figure 5A), followed by a small depolarization to about −80 mV that decayed back to the normal resting potential at the end of the current injection.

The model for the patient's muscle contained 50% WT sodium channels and 50% with L796V properties to emulate the heterozygous state. The derangements in the L796V channels were simulated as shown by the fitted curves in Figures 2–4 (see Supplementary Material for details). The predominant changes for L796V mutant channels were reduced conductance, −7.2 mV left shift of activation, and altered slow inactivation (faster kinetics, reduced voltage dependence, and less complete). The resting potential was modestly depolarized in the simulated patient muscle (−86.0 mV), and more importantly the voltage threshold for an action potential was markedly hyperpolarized at a value of −79 mV. These effects are caused by the left shift in the voltage dependence of activation for L796V channels. The same 100 ms depolarizing current injection now elicited repetitive discharges that persisted beyond the duration of the stimulus (Figure 5B). The “after-discharges” are triggered by the small depolarization produced from the use-dependent accumulation of K^+^ in the T-tubules. Each action potential produces an efflux of K^+^ into the T-tubules, and the restricted diffusion of these long narrow tubules results in an increase in extracellular [K^+^] and membrane depolarization. Normally, this small depolarization of only a few mV is insufficient to elicit action potentials, but with the left-shifted activation of L796V channels, the threshold is lower and self-sustained bursts of myotonic discharges occur.

Amongst the sodium channel gain-of-function defects known to cause myotonia, a left shift of activation is especially potent (e.g., compared to the more common cause from a slower rate of inactivation) because of the effect on action potential threshold (18, 19). Consequently, the trains of discharges tend to be very prolonged, lasting more than 10 s (Figure 5C). The mechanism by which a myotonic burst ends is not completely understood, and likely depends on several events. One proposal is that use-dependent reduction of sodium channel availability, caused by the normal trapping of channels in the slow inactivated state, reduces fiber excitability and thereby terminates the myotonic burst (20). This mechanism is impaired for L796V mutant channels because the recovery from slow inactivation is accelerated at the resting potential (Figure 4B), and the prediction is that myotonic bursts may be exceptionally prolonged. We demonstrate this effect by modifying the simulated L796V channels to include all the anomalies detected in the voltage-clamp experiments, except the kinetics of slow inactivation retained WT behavior (i.e., 3 times slower at the resting potential). Simulated muscle with this hypothetical mutant sodium channel still exhibited myotonic discharges (Figure 5D), because of the left shift of activation, but the duration of the myotonic burst was shortened. This simulation demonstrates how the addition of a slow inactivation defect can exacerbate the severity of myotonia, which heretofore has been attributed gain-of-function defects in fast gating mechanisms alone (activation and fast inactivation).

## Discussion

We report a patient with a *de novo* heterozygous *SCN4A* variant c.2386C>G; p.L796V who had congenital anomalies and early-onset myotonia. This same variant was recently been reported in a patient with multiple congenital anomalies including hip dysplasia, scoliosis, and myopathic features who developed myotonia and episodic weakness in adolescence (21). Several criteria support the assignment of pathogenic mutation to L796V. From a genetic perspective, this *de novo* heterozygous *SCN4A* allele arose independently in two families with the probands having severe myotonic syndromes with overlapping features, and the variant was not in unaffected family members or in public databases (gnomAD_v2.1.1 or ExAC, although the Moroccan population may be under represented). The L796V missense mutation is located in a functionally important transmembrane segment (S6 of domain I) that contributes to the inner vestibule of the ion-conducting pore (22). Residue L796 is highly conserved amongst human Na_V_1.x isoforms and across species. Moreover, a site three residues downstream, and therefore on the same face of the S6 helix, is an established mutation (A799S) with gain-of-function changes (23) that causes a severe myotonic phenotype with episodic laryngospasm (5). Finally, our expression studies revealed gain-of-function defects for L796V with enhanced activation and impaired slow inactivation.

The congenital onset, with secondary joint and skeletal anomalies, was notable in both L796V patients. Our patient had arthrogryposis multiplex, with the other reported L796V case having hip dysplasia and scoliosis (21). The etiology of these skeletal deformities remains to be established. In our patient, sonographic evidence of oligohydramnios was present just prior to delivery, but three prior ultrasounds (as late as 30 weeks gestation) revealed normal amniotic fluid levels. One possibility is a contribution from reduced mobility *in utero* caused by myotonia. Both L796V patients had early-onset severe myotonia, and our patient required pharmacologic intervention with sodium channel blockers to alleviate neonatal breathing difficulties. The propensity for exceptionally long-duration myotonic discharges in our model simulation with the L796V functional defects (Figure 5) may predispose to secondary joint defects. A monoallelic variant of HSPG2 was identified in our patient, and while we cannot exclude the possibility of a modifier effect that exacerbates myotonia from the sodium channel L796V defect, we think this is unlikely for several reasons. First, perlecan staining was normal. Second, the needle EMG at 6 weeks of age (Supplementary Video 2) showed classical myotonic runs that waxed and waned in frequency and amplitude. Conversely, in the Schwartz-Jampel syndrome (SJS) with proven biallelic mutations of HSPG2 the needle EMG shows complex repetitive discharges of constant amplitude and frequency, with abrupt discontinuation of the burst (24). These discharges in SJS are attributed to peripheral nerve hyperexcitability, rather than myotonia from altered sarcolemmal excitability. Third, our model simulations show the functional defect of L796V alone is sufficient to cause exceptionally prolonged myotonic bursts. Congenital joint and bone deformities are not a frequent accompaniment of congenital myotonia, but have been reported. Club foot with peripheral contractures, hip dislocation, and facial dysmorphism were reported for a newborn with diffuse muscle stiffness and widespread myotonic discharges who later developed muscle hypertrophy and was found to be heterozygous for *SCN4A* c.3539A>T; p.N1180I (25). Severe scoliosis with peripheral contractures in childhood was described for siblings with myotonic stiffness, profuse myotonic discharges, and the *SCN4A* p.P1158A mutation (26), but no details were provided about the clinical presentation at birth.

Congenital myopathy was a feature of the prior report for L796V, as manifest by polyhydramnios, fetal hypokinesia, hip dysplasia, and later progression to include high arched palate and elongated face (21). A muscle biopsy at age 27 had myopathic features with type I fiber predominance and hypertrophy. The other case of congenital myotonia with joint abnormalities (*SCN4A* p.N1180I) also had signs of neonatal myopathic weakness with polyhydramnios, high arched palate, and downslanting palpebral fissures (25). These cases are distinctly different, however, from the recently described syndrome of congenital myopathy with severe fetal hypokinesia caused by biallelic mutations for *SCN4A* (15, 27). In this latter syndrome, the core phenotype includes neonatal hypotonia, moderate to severe myopathic weakness that may be fatal, and *SCN4A* loss-of-function mutations that are asymptomatic in heterozygous parents. In contrast, the heterozygous p.L796V patients and the p.N1108I case had neonatal myotonic stiffness, only mild myopathic weakness that subsequently improved and in some progressed to muscle hypertrophy, and for p.L796V, experimentally established gain-of-function defects. Electromyographic evidence of myotonia in early infancy, as we observed at 6 weeks, is unusual and differentiates the weakness in our patient from the syndrome of congenital myopathy with hypotonia associated with recessive loss-of-function mutations of *SCN4A* (15). Myopathic features may be a component of the dominantly inherited *SCN4A* myotonic syndromes (sodium-channel myotonia, myotonia permanens, severe neonatal episodic laryngospasm, SNEL) or the late permanent muscle weakness of periodic paralysis. In our view, however, neither a single allelic variant of *SCN4A* nor a dominant inheritance pattern has been associated with a syndrome for which congenital myopathy is the predominant feature.

**Figure 6:**
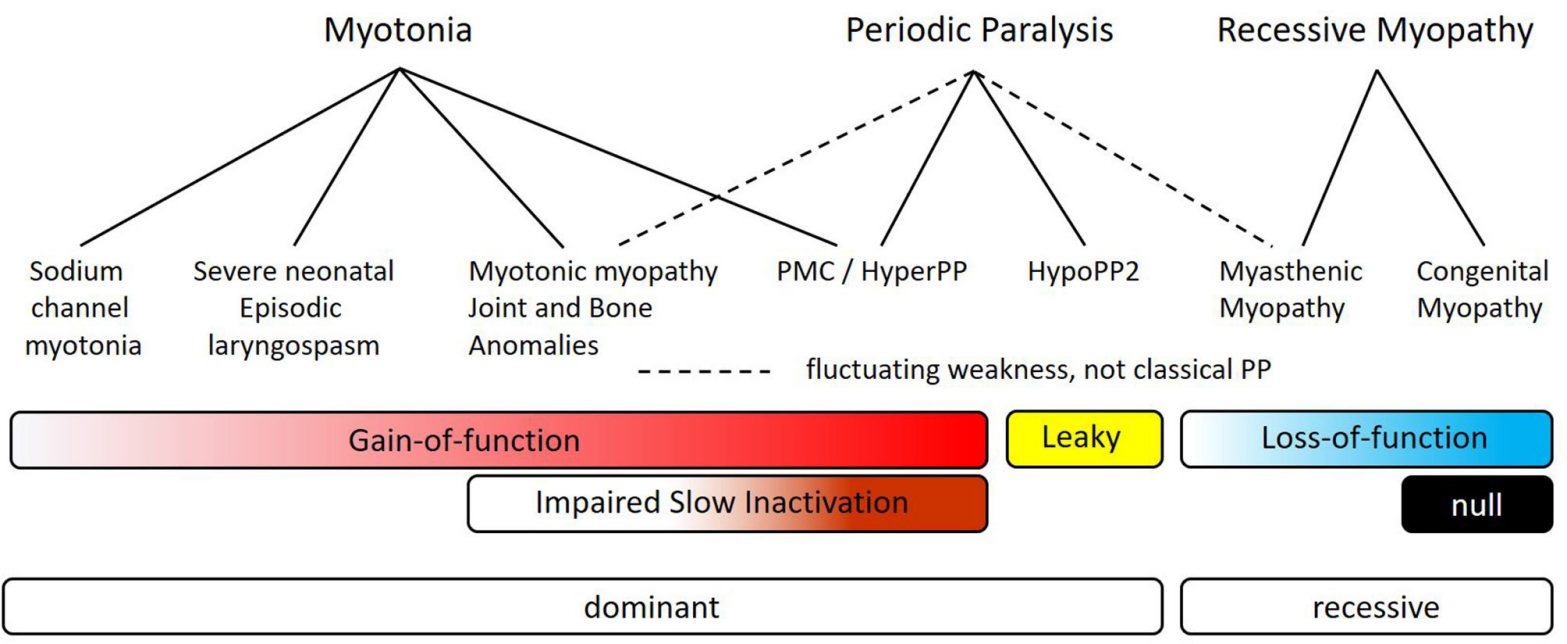
Myotonic myopathy with secondary joint and bone anomalies is a dominantly expressed allelic disorder on the spectrum of *SCN4A* sodium channelopathies of skeletal muscle. The top row depicts the primary clinical manifestations, which may be overlapping in specific disorders (second row). Dashed lines are used to indicate fluctuating muscle weakness for myotonic myopathy and for myasthenic myopathy, as distinct from the more clearly demarcated attacks of classical periodic paralysis that usually have well-defined trigger factors (solid lines). The bars show the overlap of different functional defects of mutant sodium channels, with the more intense color indicating more severe changes. The final row indicates the inheritance pattern for expression of symptoms.

We propose a new *SCN4A* syndrome, myotonic myopathy with secondary joint and bone anomalies, should be applied to the phenotype for p.L796V and p.N1108I. The core elements are congenital joint and bone anomalies with neonatal or infantile myotonic stiffness and widespread myotonic discharges. Breathing difficulties may be present, as in our patient, but stridor and respiratory compromise are not the predominant presentation. The relation of myotonic myopathy with joint and bone anomalies to the other sodium channelopathies of skeletal muscle is illustrated in Figure 6. This new syndrome is envisioned to be positioned between SNEL and paramyotonia congenita (PMC). Overlap with SNEL may be manifest as breathing difficulties and with PMC as episodic weakness [reported for p.L796V (21)]. We have previously shown that impairment of slow inactivation predisposes to episodes of periodic paralysis, and the slow inactivation defect observed herein for L796V may account for susceptibility to episodic weakness.

## Data Availability Statement

The datasets generated for this study can be found in the National Center for Biotechnology Information. ClinVar; [VCV000383923.3], https://www.ncbi.nlm.nih.gov/clinvar/variation/VCV000383923.3 (accessed December 5, 2019).

## Ethics Statement

The research protocol was approved by the Children's Hospital of Eastern Ontario Research Ethics Board, and clinical data were obtained in a manner conforming with research ethics board and funding agency guidelines. Written informed consent to participate in this study was provided by the participants' legal guardian/next of kin.

## Author Contributions

NE, SC, GG, and HM designed the study. HM performed the clinical assessment. TN, LH, and GG performed the genetic analysis. NE measured sodium currents and analyzed them with SC. Computer simulations were performed by SC. SC, GG, and HM wrote the paper.

### Conflict of Interest

The authors declare that the research was conducted in the absence of any commercial or financial relationships that could be construed as a potential conflict of interest.

## Supplementary Material

Supplementary Video 1 EMG at 2 weeks of age shows increased insertional activity.

Supplementary Video 2 EMG at 6 weeks of age shows myotonia.
