# Corrigendum: Myotonic Myopathy With Secondary Joint and Skeletal Anomalies From the c.2386C>G, p.L796V Mutation in *SCN4A*

**DOI:** 10.3389/fneur.2020.00181

**Published:** 2020-03-20

**Authors:** Nathaniel Elia, Trystan Nault, Hugh J. McMillan, Gail E. Graham, Lijia Huang, Stephen C. Cannon

**Affiliations:** ^1^Department of Physiology, David Geffen School of Medicine at UCLA, Los Angeles, CA, United States; ^2^Molecular, Cellular, and Integrative Physiology Program, UCLA, Los Angeles, CA, United States; ^3^Division of Neurology, Children's Hospital of Eastern Ontario, University of Ottawa, Ottawa, ON, Canada; ^4^Department of Genetics, Children's Hospital of Eastern Ontario, University of Ottawa, Ottawa, ON, Canada

**Keywords:** skeletal muscle, channelopathy, sodium channel, Na_V_1.4, myotonia, voltage-clamp

In the original article there was a typographical error in the number for the amino acid missense mutation in *SCN4A*. The correct designation is L796V, which was erroneously transposed as L769V. Corrections have been made in several sections of the paper.

The title, which now reads:

“Myotonic Myopathy With Secondary Joint and Skeletal Anomalies From the c.2386C>G, p.L796V Mutation in *SCN4A*.”

The Abstract:

“The phenotypic spectrum associated with the skeletal muscle voltage-gated sodium channel gene (*SCN4A*) has expanded with advancements in genetic testing. Autosomal dominant *SCN4A* mutations were first linked to hyperkalemic periodic paralysis, then subsequently included paramyotonia congenita, several variants of myotonia, and finally hypokalemic periodic paralysis. Biallelic recessive mutations were later identified in myasthenic myopathy and in infants showing a severe congenital myopathy with hypotonia. We report a patient with a pathogenic *de novo SCN4A* variant, c.2386C>G p.L796V at a highly conserved leucine. The phenotype was manifest at birth with arthrogryposis multiplex congenita, severe episodes of bronchospasm that responded immediately to carbamazepine therapy, and electromyographic evidence of widespread myotonia. Another *de novo* case of p.L796V has been reported with hip dysplasia, scoliosis, myopathy, and later paramyotonia. Expression studies of L796V mutant channels showed predominantly gain-of-function changes, that included defects of slow inactivation. Computer simulations of muscle excitability reveal a strong predisposition to myotonia with exceptionally prolonged bursts of discharges, when the L796V defects are included. We propose L796V is a pathogenic variant, that along with other cases in the literature, defines a new dominant *SCN4A* disorder of myotonic myopathy with secondary congenital joint and skeletal involvement.”

The final paragraph of the Introduction:

“We report a patient who presented with arthrogryposis multiplex congenita, congenital myopathy, and episodes of bronchospasm who has the c.2386C>G, p.L796V variant in *SCN4A*. Expression studies of L796V channels revealed a two forms of gain-of-function, enhanced activation and impaired slow inactivation, and which in model simulations led to prolonged bursts of myotonic discharges. We propose L796V is a pathogenic mutation and that the clinical features shared with previously described cases defines a new *SCN4A* syndrome of myotonic myopathy with secondary deformities of joints and bone.”

The Results section, subsection Functional Characterization of L796V Mutant Sodium Channels, sub-subsection Slow Inactivation Was Impaired by L796V, final paragraph:

“At first glance, the changes in slow inactivation properties for L796V channels appear to be a mixture of gain and loss of function effects. Enhancement of slow inactivation is expected at the resting potential of −80 mV because of the reduced slope of the voltage dependence and the tendency for a left shift (Figure 3D, reduced availability at −80 mV), as well as for a faster rate of entry over the voltage range of −50 to −30 mV (Figure 3C, smaller time constants). On the other hand, impairment of slow inactivation is expected at depolarized potentials because inactivation of L796V is less complete than WT (Figure 3D, higher plateau −20 to 20 mV), and the recovery from slow inactivation is faster for L796V at the resting potential (Figure 3C, smaller time constant at −80 mV). We propose the overall effect will be impaired slow inactivation for L796V channels, in the context of the slow inactivation that occurs during sustained bursts of action potentials (e.g., myotonia). The basis for this prediction is that entry to slow inactivation occurs primarily at voltages near the peak depolarization of the action potential (where the predominant change is less complete slow inaction for L796V) and trapping of channels in the slow inactivated state is primarily dependent on the rate of recovery at the resting potential of −80 mV (which is faster for L796V channels). This prediction is supported by experimental evidence showing the use-dependent reduction of sodium current is more pronounced for WT than L796V channels during repetitive stimulation at 50 Hz. Figure 4A shows a superposition of sodium currents recorded in response to the first 10 pulses to +10 mV from a holding potential of −80 mV. The initial decline in peak amplitude from the first to the second pulse is predominantly caused by incomplete recovery from fast inactivation, whereas the subsequent decline for additional pulses is due to progressive loss of channel availability from slow inactivation. The slow inactivation effect is illustrated in Figure 4B for the entire 40 second train of 3 ms depolarizations at 50 Hz (2,000 pulses). The peak amplitude for each pulse is normalized by the amplitude of the second pulse (Figure 4A, blue trace) to isolate the effect of slow inactivation, which under these conditions is about 10% less for L796V channels compared to WT.”

The legend to Figure 1:

“Figure 1. Activation of L796V channels is shifted toward more negative potentials. Sodium currents were recorded from HEK cells expressing WT **(A)** and L796V **(B)** channels. Superimposed traces show currents elicited by depolarization to test potentials of −75 to +60 mV from a holding potential of −120 mV. **(C)** Peak sodium current is shown as a function of test potential and reveals a reduced amplitude for L796V compared to WT. **(D)** Transforming the peak current to relative conductance (see Methods and Materials) shows a 7.2 mV hyperpolarized shift for L796V channels. Symbols show means from *n* = 16 (WT) or *n* = 10 (L796V) cells.”

The legend to Figure 5:

“Figure 5. Model simulation predicts sustained busts of myotonia from the L796V channel defects. **(A)** The action potential elicited by a 20 μA/cm^2^ current pulse applied for 100 ms is shown for a simulated muscle fiber with normal values for voltage-activated ion channels. Inset shows the initial 25 ms of the model simulation. **(B)** When 50% of the simulated sodium channels are modeled using parameters to emulate the altered behavior of L796V channels, then the same 100 ms current stimulus triggers a burst of myotonic discharges that persist beyond the 100 ms duration of the stimulus. **(C)** Extended simulation over time shows stable self-sustained repetitive myotonic discharges that do not cease. **(D)** When the simulated mutant channels are modified to have the slow inactivation kinetics for WT channels, then use-dependent reduction of the sodium current is enhanced (see this figure) and the myotonic burst ends after 4.5 s.”

The authors apologize for this error and state that this does not change the scientific conclusions of the article in any way. The original article has been updated.

