# Supplementary material for: Myotonic Myopathy With Secondary Joint and Skeletal Anomalies From the c.2386C>G, p.L796V Mutation in SCN4A
Source: Front Neurol. 2020 Feb 13;11:77. doi: 10.3389/fneur.2020.00077 (PMC7031655; doi:10.3389/fneur.2020.00077)
Supplement: Supplementary file 1 [file Table_1.DOCX]

**Supplementary Material**

**Simulation of L796V sodium channel gating defects**

The gating behavior for WT and L796V sodium channels was simulated with a modified version of the Hodgkin-Huxley equations. The sodium current, $I_{Na}(V,t)$ , was modeled as a combination of activation, $m(V,t)$ , fast inactivation, $h(V,t)$ , and slow inactivation, $s(V,t)$ , that collectively determined the open probability of the channel, and $i_{Na}(V)$ was the voltage dependence of current flow through an open channel.

$I_{Na}\left( V,t \right)= {m\left( V,t \right)}^{3}h\left( V,t \right)s(V,t)i_{Na}(V)$ .

Each of the gating terms ($m, h, s$) was approximated by a single transition between two states. For example, channel activation was modeled as:

$$closed "1 - m" \begin{matrix} \underset{\to}{\alpha_{m}\left( V \right)} \\ \overset{\leftarrow}{\beta_{m}\left( V \right)} \end{matrix} open "m"$$

such that

$\frac{dm}{dt}= \alpha_{m}\left( 1-m \right)- \beta_{m}m$ .

The parameter estimates for the voltage-dependent rate constants, $\alpha_{m}(V)$ and $\beta_{m}(V)$, were obtained by fitting the voltage-clamp data for sodium currents in HEK cells. The reduced data are represented as voltage-dependent time constants, $\tau_{m}(V)$ , and steady-state probability, $m_{\infty}(V)$, for which the rate constant dependencies are:

$\tau_{m}= 1/{(\alpha_{m}+ \beta_{m})}$ and $m_{\infty}= {\alpha_{m}}/{(\alpha_{m}+ \beta_{m})}$

Empirically, we fit the steady-state data to a Boltzmann equation (see Methods) and then determined the parameters for the rate constants by non-linear least-square fit (Origin 9.1, Microcal) to

$\alpha_{m}= {m_{\infty}}/{t_{m}}$ and $\beta_{m}= {(1-m_{\infty})}/{\tau_{m}}$ .

This same procedure was used to estimate the parameter values for the rate constants governing fast inactivation and slow inactivation. The rate equations were similar to those used by Hodgkin and Huxley:

$\alpha_{m}= {\overline{\alpha_{m}}(V-V_{m})}/{(1- e^{-\left( V-V_{m} \right){/k}_{\alpha m}})}$ $\beta_{m}= \overline{\beta_{m}} e^{-(V-V_{m)/k_{\beta m}}}$

$\alpha_{h}= {\overline{\alpha_{h}}}/{(1+ e^{\left( V-V_{\alpha h} \right){/k}_{\alpha h}})}$ $\beta_{h}= {\overline{\beta_{h}}}/{{(1+ e}^{-(V-V_{\beta h)/k_{\beta h}}})}$

$\alpha_{s}= \overline{\alpha_{s}} e^{-V/k_{\alpha s}}$ $\beta_{s}= {\overline{\beta_{s}}}/{{(1+ e}^{-(V-V_{s)/k_{\beta s}}})}$

The estimated parameter values for WT and L796V channels are listed in Table S1. In the computer simulation of a muscle fiber (see below), the gating terms were computed from the independent fits to the voltage-dependent time constant (using $\alpha\left( V \right) \text{and} \beta(V)$ as above) and to the steady-state probability (using a Boltzmann function). For example, the activation term, $m$, was computed as

$$\frac{dm}{dt} = \frac{m_{\infty}-m}{\tau_{m}} .$$

| Table S1 Parameters for sodium channel gating variables: $m, h, s$ | | | | |
| --- | --- | --- | --- | --- |
| Gating | Parameter | Units | WT | L796V |
|  |  |  |  |  |
| Activation, $m$ | $\overline{\alpha_{m}}$ ^1^ | msec^-1^ | 0.28 | 0.28 |
|  | $V_{m}$ | mV | -46 | -46 |
|  | $k_{\alpha m}$ | mV | 10 | 10 |
|  | $\overline{\beta_{m}}$ | msec^-1^ | 1.4 | 1.4 |
|  | $k_{\beta m}$ | mV | 18 | 18 |
|  | $V_{1/2}$ ^2^ | mV | -46 | -53.2 |
|  | $k$ ^3^ | mV | 4.3 | 4.9 |
|  |  |  |  |  |
| Fast Inactivation, $h$ | $\overline{\alpha_{h}}$ | msec^-1^ | 0.51 | 0.63 |
|  | $V_{\alpha h}$ ^2^ | mV | -110 | -117 |
|  | $k_{\alpha h}$ | mV | 10 | 12 |
|  | $\overline{\beta_{h}}$ | msec^-1^ | 3.3 | 2.9 |
|  | $V_{\beta h}$ ^2^ | mV | -26 | -26 |
|  | $k_{\beta h}$ | mV | 9.1 | 11 |
|  | $V_{1/2}$ ^2^ | mV | -75 | -77 |
|  | $k$ ^3^ | mV | 5.4 | 5.0 |
|  |  |  |  |  |
| Slow Inactivation, $s$ | $\overline{\alpha_{s}}$ | msec^-1^ | 4.0 x 10^-4^ | 3.7 x 10^-4^ |
|  | $k_{\alpha s}$ | mV | 21 | 230 |
|  | $\overline{\beta_{s}}$ | msec^-1^ | 7.3 x 10^-4^ | 1.3 x 10^-4^ |
|  | $V_{\beta s}$ | mV | -17 | -7.4 |
|  | $k_{\beta s}$ | mV | 21 | 11 |
|  | $V_{1/2}$ | mV | -59 | -67 |
|  | $k$ | mV | 11 | 14 |
|  | $s_{0}$ | - | .012 | 0.23 |
| 1 Values for parameters of activation kinetics ($\overline{\alpha_{m}}, V_{m}, k_{\alpha m}, \overline{\beta_{m}}, k_{\beta m}$) taken from Cannon et al. (1993).  2 Value for WT has a hyperpolarized shift, to account for differences between HEK cells and mammalian muscle (Fu *et al.*, 2011). Relative shift for L796V was set to the value from this study.  3 Value for WT was set to the steepness of voltage-dependence observed in mammalian muscle (Fu *et al.* , 2011). Ratio of ${k_{WT}}/{k_{L796V}}$ was set to the value from this study.  The most impactful parameter changes between WT and L796V that contribute to sustained myotonic discharges are highlighted in blue. | | | | |

**Simulation of muscle fiber electrical excitability**

The simulated muscle fiber is an extension of our two compartment model (Cannon *et al.* , 1993), that represents the sarcolemma and transverse tubular (T-tubule) membranes. For our simulated fiber with a radius of 40 μm, the ratio of T-tubule membrane surface area to sarcolemmal membrane surface area is $\gamma$ = 4.8, separated by an access resistance of $R_{a}$= 40 Ω-cm^2^. Each membrane compartment contains a voltage-dependent sodium conductance and a delayed rectifier potassium conductance as before (Cannon *et al.* , 1993). The revised model herein also includes an inward rectifier potassium conductance (Struyk and Cannon, 2008), and a voltage-activated chloride conductance (DiFranco *et al.*, 2011) replaces the “leak” conductance in the prior model. The open-channel current-voltage relation for each conductance was simulated using the GHK constant field equation (Hille, 2001). Finally, a pump current was included to simulate the electrogenic contribution from the Na/K-ATPase (Wallinga *et al.*, 1999).

The total membrane current per unit area of sarcolemma, $I_{m}$ , is a combination capacitive current, ionic currents through conductances and pumps, plus the net current from the T-tubule:

$$I_{m}= C_{m}\frac{dV}{dt}+ \sum I_{ionic} + I_{pump} + I_{TT}$$

where

$$\sum I_{ionic}= I_{Na}+ I_{Kir}+ I_{Kdr}+ I_{Cl} \text{and}\text{ } I_{TT}= \frac{V- V_{TT}}{R_{a}} .$$

Similarly, the total current across the T-tubule (per unit area of sarcolemmal membrane) is

$$I_{TT}= \gamma C_{m}\frac{dV_{TT}}{dt}+ \gamma\sum I_{TT\_ionic} .$$

The concentration of K^+^ in the T-tubule (an extracellular space) increases in response to the firing of action potentials (Cannon *et al.* , 1993, Wallinga *et al.* , 1999), which produce an efflux of myoplasmic K^+^ during repolarization. This increase is balanced by losses from influx through the Na/K-ATPase and from equilibration with the interstitial space (passive diffusion). The net mass balance is

$$\frac{d{[K]}_{TT}}{dt}=\frac{\left( I_{TT_{Kir}}+ I_{TT_{Kdr}}- I_{TT_{pump K}} \right)}{F\varsigma} - \frac{\left[ K \right]_{TT}- \left[ K \right]_{out}}{\tau_{K}}$$

where $\varsigma$ = 10^-6^ cm is the volume to surface area ratio of the T-tubule, and $\tau_{K}$ = 350 msec is the decay time constant for equilibration of T-tubule [K]_TT_ with the interstitial [K]_out_.

These coupled differential equations were solved numerically by Euler integration with an adaptive step size, $\Delta t$, such that the change in membrane potential for a single step was less than 0.05 mV. Parameter values for the voltage-dependent gating of ionic conductances were set to the values in the references cited above, and the maximum permeability for the conductances in each membrane compartment are listed in Table S2. Ion concentrations for each compartment are listed in Table S3.

| Table S2. Maximum permeability (cm/sec) | | |
| --- | --- | --- |
| channel type | sarcolemma | T-tubule |
| P_Na_WT_ | 1.1 x 10^-4^ | 0.88 x 10^-4^ |
| P_Na_L796V_ | 0.81 x 10^-4^ | 0.65 x 10^-4^ |
| P_Kir_ | 6.4 x 10^-6^ | 6.4 x 10^-6^ |
| P_Kdr_ | 2.4 x 10^-5^ | 2.4 x 10^-5^ |
| P_Cl_ | 8.0 x 10^-4^ | 2.4 x 10^-4^ |

| Table S3. Ion Concentrations (mM) | | | |
| --- | --- | --- | --- |
| Ion | myoplasm | T-tubule | interstium |
| Na^+^ | 15.1 | 140 | 140 |
| K^+^ | 152 | 4.0 * | 4.0 |
| Cl^-^ | 4.0 | 105 | 105 |
| * concentration varies with simulated fiber electrical activity | | | |

**References**

Cannon SC, Brown RH, Jr., Corey DP. Theoretical reconstruction of myotonia and paralysis caused by incomplete inactivation of sodium channels. Biophys J. 1993;65(1):270-88.

DiFranco M, Herrera A, Vergara JL. Chloride currents from the transverse tubular system in adult mammalian skeletal muscle fibers. J Gen Physiol. 2011 Jan;137(1):21-41.

Fu Y, Struyk A, Markin V, Cannon S. Gating behaviour of sodium currents in adult mouse muscle recorded with an improved two-electrode voltage clamp. J Physiol. 2011 Feb 1;589(Pt 3):525-46.

Hille B. Ion Channels of Excitable Membranes. 3rd ed. Sunderland, MA: Sinauer; 2001.

Struyk AF, Cannon SC. Paradoxical depolarization of Ba^2+^- treated muscle exposed to low extracellular K^+^: insights into resting potential abnormalities in hypokalemic paralysis. Muscle Nerve. 2008 Mar;37(3):326-37.

Wallinga W, Meijer SL, Alberink MJ, Vliek M, Wienk ED, Ypey DL. Modelling action potentials and membrane currents of mammalian skeletal muscle fibres in coherence with potassium concentration changes in the T-tubular system. Eur Biophys J. 1999;28(4):317-29.
